# Ethics in practice: the state of the debate on promoting the social value of global health research in resource poor settings particularly Africa

**DOI:** 10.1186/1472-6939-12-22

**Published:** 2011-11-15

**Authors:** Geoffrey M Lairumbi, Parker Michael, Raymond Fitzpatrick, Michael C English

**Affiliations:** 1 Child and Newborn Health Group, Kemri-Wellcome Trust Research Programme, P.O Box 43640, Nairobi, Kenya; 2 Ethox Centre, Department of Public Health, Oxford University, Old Rd Campus, Headington, Oxford, OX3 7LF, UK; 3 Department of Public Health, Oxford University, Old Rd Campus, Headington, Oxford, OX3 7LF, UK; 4 Department of Paediatrics, University of Oxford, Level 2, Children's Hospital, John Radcliffe, Headington, Oxford, OX3 9DU, UK

## Abstract

**Background:**

Promoting the social value of global health research undertaken in resource poor settings has become a key concern in global research ethics. The consideration for benefit sharing, which concerns the elucidation of what if anything, is owed to participants, their communities and host nations that take part in such research, and the obligations of researchers involved, is one of the main strategies used for promoting social value of research. In the last decade however, there has been intense debate within academic bioethics literature seeking to define the benefits, the beneficiaries, and the scope of obligations for providing these benefits. Although this debate may be indicative of willingness at the international level to engage with the responsibilities of researchers involved in global health research, it remains unclear which forms of benefits or beneficiaries should be considered. International and local research ethics guidelines are reviewed here to delineate the guidance they provide.

**Methods:**

We reviewed documents selected from the international compilation of research ethics guidelines by the Office for Human Research Protections under the US Department of Health and Human Services.

**Results:**

Access to interventions being researched, the provision of unavailable health care, capacity building for individuals and institutions, support to health care systems and access to medical and public health interventions proven effective, are the commonly recommended forms of benefits. The beneficiaries are volunteers, disease or illness affected communities and the population in general. Interestingly however, there is a divide between "global opinion" and the views of particular countries within resource poor settings as made explicit by differences in emphasis regarding the potential benefits and the beneficiaries.

**Conclusion:**

Although in theory benefit sharing is widely accepted as one of the means for promoting the social value of international collaborative health research, there is less agreement amongst major guidelines on the specific responsibilities of researchers over what is ethical in promoting the social value of research. Lack of consensus might have practical implications for efforts aimed at enhancing the social value of global health research undertaken in resource poor settings. Further developments in global research ethics require more reflection, paying attention to the practical realities of implementing the ethical principles in real world context.

## Background

The social value of international health related research at a minimum refers to efforts aimed at ensuring global health research contributes to improvements in human health, through for instance the generation and application of generalisable knowledge [1], and has recently become a key concern in global research ethics, especially when this research is undertaken in resource poor settings. Various strategies have been devised to promote the social value of research including the call for benefit sharing. This entails the elucidation of what if anything, is owed to participants, their communities and host nations that take part in such research. Other strategies include development of partnerships to identify priorities for research [2] and community engagement to enhance protection of research participants [3]. These developments have further been crystallised through research ethics guidelines that should provide direction when considering benefit sharing. However, debates continue in the academic bioethics literature relating to defining benefits, beneficiaries, and the scope of obligations for providing benefits.

Debate over which benefits are ethically justifiable show a division of opinion between those favouring determination based on the type of research [4] and those calling for a generic approach based on background issues of exploitation and oppression that produce ill health within resource poor settings [5-7]. Those in favour of the community as the appropriate beneficiary, hold the view that providing benefits to only those participating in research does not protect communities against exploitation, because it fails to engage with the wider question of justice that connects community health needs and the conditions of poverty that characterise resource poor settings [7-10]. In contrast, those in favour of benefiting individual participants maintain that research does not and should not have a primary aim of restoring inequalities or providing absent services, and should therefore not be subject to the principles guiding health care provision [11-13]. Debates relating to obligations for providing benefits mainly address the appropriate limit or the scope of the benefits both during the course of research (ancillary care responsibilities) and after completion (post trial obligations). The debates are mainly articulated by making reference to who should be the beneficiary. Some people suggest that both sponsors and governments should be held responsible, especially for research studies aimed at wide deployment of interventions addressing population health [14-16], while others recommend development of collective responsibility through partnerships, especially when the anticipated benefits have huge financial implications [17-20].

While these debates may be indicative of willingness at the international level to engage with such ethical issues in global health research it remains unclear which position among those advanced in the literature should be adopted, and how such positions should be justified in ethical terms. The work reported here examines the normative recommendations from various research ethics guidelines regarding these responsibilities, as part of a broader project examining the practical aspects of promoting the social value of international collaborative health related research undertaken in a resource poor setting.

## Methods

We reviewed documents selected from the international compilation of research ethics guidelines by the Office for Human Research Protections under the US Department of Health and Human Services [21]. The compilation contains 1,100 laws, regulations and guidelines governing human subjects' research in 96 countries worldwide. These laws and regulations are categorised into those that provide general guidance to all human subjects' research and those that are specific to drugs, genetic research, human biological materials, data protection/privacy and stem cell research.

The selection criteria were based on the relevance of the guideline as sources of ethical guidance for research related to health conducted in resource poor settings. Relevance was determined on the basis of the jurisdiction and the type of research covered. Research ethics guidelines with a wider mandate in the sense that they were expected to provide guidance to researchers in the USA and Europe while conducting research in developing countries were included. The inclusion criteria were limited to these regions since a high proportion of health related research that is undertaken in Kenya, our country of particular focus, involves collaboration with researchers and institutions from Europe and the Americas [22]. Although it is acknowledged that there are resource poor countries in other continents, the focus within this study, were those in Africa.

Guidelines that provide general guidance for all types of health related research, and those aimed at specific types of clinical research^1 ^such as drug or vaccine trials or general epidemiological studies including genetic research were selected. This selection criterion was based on the fact that much of the health related research conducted in resource poor settings are drug or vaccine trials and general epidemiological studies. In terms of the geographical jurisdiction, guidelines/documents specific to the African region were identified from the OHRP list. Due to the smaller number of guidelines available online through the OHRP all the 7 out of 14 guidelines from African countries were reviewed. The guidelines from Egypt, Botswana, The Gambia and Malawi were not available online and attempts to obtain them by writing to authors were not successful. In addition guidelines from Brazil and India were deliberately included in the review, due to the fact that some of the initial Perinatal HIV Transmission Trials [23,24] that triggered the fair benefits debates in global health research were conducted in these countries. While the international compilation of guidelines by the OHRP is not exhaustive, further literature search to identify additional country specific guidelines was not successful^2^.

Based on theoretical literature on researcher's responsibilities in global health research a data abstraction form was developed to provide a systematic framework for recording information from the guidelines relating to specific clauses that addressed responsibilities in terms of the benefits, the beneficiaries, obligations and mechanisms through which such decisions are made. The information extracted from these documents were organised into tables that were used in shedding light on the nature of recommendations given by different research ethics guidelines.

## Results

### Description of the guidelines reviewed

The research ethics guidelines that were reviewed and a brief contextual background on their jurisdiction are presented in Additional file 1. The general guidelines with a global jurisdiction that were reviewed include; i) the Declaration of Helsinki by the World Medical Association (WMA), ii) the international guidelines for biomedical research involving human subjects by the International Organisation for Medical Sciences (CIOMS), iii) the guidelines for good clinical practice (GCP) promulgated by the International Conference on Harmonisation (ICH), iv) the Report on the Ethical and Policy Issues relating to the conduct of Research Involving Human Participants by the US National Bioethics Advisory Committee (NBAC), v) the statement on benefit sharing by the Human Genome Organisation (HUGO), vi) the report on the Ethical Aspects of Clinical Research in Developing Countries by the European Group on Ethics (EGE), vii) the Universal Declaration on Bioethics and Human Rights (UDBHR) by United Nations Educational Social and Cultural Organisation (UNESCO), viii) the Report on the Ethics of Research Related to health care in developing countries by the Nuffield Council on Bioethics (NCOB) and the guidance document on Ethical Considerations in biomedical HIV prevention trial by the UNAIDS/WHO. Country-specific guidelines that were included are those from Kenya, Uganda, South Africa, Nigeria, Sudan, Zimbabwe, Ethiopia, India and Brazil.

### The need to consider benefit sharing

Close to half of the guidelines reviewed have specific articles/clauses recommending consideration of the value of research, or pointing to the need to engage with such a consideration in general terms. Guidelines were examined for particular clauses that made specific mention of the responsibilities of researchers in relation to the benefits, the beneficiaries, obligations and the mechanisms through which such decisions should be made. Table 1 shows the specific clauses identified within all the guidelines that address such responsibilities.

**Table 1:** Exemplars of clauses supporting the need to consider research benefits^1^.

***Guidelines Code/Report***	***Relevant clause***
**Declaration of Helsinki (2008)**	Guideline 5 "In medical research on human subjects, considerations related to the wellbeing of the human subject should take precedence over those of science and society" Guideline 19 "Medical research is only justified if there is reasonable likelihood that the population in which it is carried out stand to benefit".

**CIOMS (2002)**	Guidelines 5, 10, 11 and 12 require that information regarding benefits to participants, the community and how and when these benefits shall be made available, be given during the consent process.

**NBAC (2004)**	Part 4.2 states that "whenever possible/.../agreements should be negotiated/.../to make effective interventions or other research benefits, available to the host country",

**ICH-GCP(1996)**	Part 4.3.2 states that "During and following a subject's participation in a trial, the investigator/institution should ensure that adequate medical care is provided to a subject for any adverse events, including clinically significant laboratory values, related to the trial.../.

**UDBHR 2005**	Article 15 "the benefits resulting from any scientific research and its applications should be shared..."

**Nigerian Ethics Code (2006)**	Section A, "research must have social and scientific value to either participants or the population that they represent, the local community, host country or the world, in order to justify the use of finite resources and exposure to harm/.../in collaborative studies...",

**Brazilian Resolution No. 196/96**	Section III: part 3 M, "...research protocols should guarantee the individuals and communities where the research was undertaken, a return on the benefits obtained in the research/.../when it is really beneficial to foster or encourage changes in practices/behaviors in the interest of a community..."

### What forms of benefit sharing are recommended?

In most cases, the guidance relating to what benefits can potentially be provided are articulated within a continuum of time when research is being undertaken. Accordingly, research benefits are articulated as either those that are concurrent to the conduct of research or those that are expected after the research is complete. Table 2 presents the forms of benefits that are recommended by international and regional specific research guidelines.

**Table 2:** Classification of forms of benefit sharing.

Research Ethics Guidelines/Report/Code	Form of Benefit sharing				
	
	***Access to Unproven Interventions***	***Provide H/care***	***Capacity Building***	***Support to health system***	***Post Trial Access***
Declaration of Helsinki (WMA)2008	✓				✓

Council for international Organisation for Medical Sciences(CIOMS) 2002	✓		✓		✓

Council for international Organisation for Medical Sciences (Epidemiology) 2007		✓			✓

National Bioethics Advisory Commission Report 2001	✓		✓		✓

Nuffield Council on Bioethics Report 2005			✓		✓

International Conference on Harmonisation --(GCP) 1996	✓				

Council of Europe 2003	✓				✓

HUGO Statement on Benefit Sharing 2000		✓	✓		

UNESCO Declaration on Bioethics and Human Rights 2005	✓	✓	✓	✓	✓

UNAIDS 2007		✓	✓		

Guidelines by the |Kenya National Council for Science & Technology 2004			✓		✓

Ethiopian National Health Research Ethics Guideline(2005)		✓			✓

National Guidelines for Ethical Conduct of Research Involving Human Subjects by the Sudanese National Ministry of Health (Directorate General of Health Planning and Research)	✓	✓			✓

Guidelines for researchers and Ethics Review Committees in Zimbabwe (Medical Research Council of Zimbabwe) (2004)	✓				✓

Ugandan Guidelines by the National Council for Science & Technology 2006			✓		✓

S. Africa MRC (2005)				✓	

Nigeria National Code of Health Research(2006)		✓	✓	✓	

Brazil 1996	✓	✓			✓

India Council on Medical Research (ICMR) Code 2000		✓	✓	✓	

Table 3 presents examples of benefits which may be considered while negotiating benefit sharing both during and after research is complete. In the case of benefits while research is on-going, access to interventions being researched is recommended by most guidelines as a key benefit by most of the guidelines. Other forms of benefits include provision of unavailable health care, capacity building for individuals and institutions, support to health care systems and access to medical and public health interventions once they are proven effective.

**Table 3:** Class of beneficiaries within research ethics guidelines.

	Category of Beneficiaries		
	
Guidelines/Report/Code	***Volunteers/individual Participants***	***Trial/Disease Community Res. Inst/System***	***Larger Community/Host country***
**Declaration of Helsinki (2008)**	✓		

**Council for international Organisation for Medical Sciences (2002)**	✓		

**Council for international Organisation for Medical Sciences (Epidemiology) (2009)**		✓	

**National Bioethics Advisory Commission Report (2001)**	✓	✓	

**Council of Europe 2003**	✓	✓	

**Nuffield Council on Bioethics (2005)**	✓		

**HUGO Statement on Benefit Sharing (2000)**	✓	✓	

**UNESCO Declaration on Bioethics and Human Rights (2005)**	✓	✓	✓

**UNESCO Declaration on Human Genome and Human Rights (1997)**			✓

**UNAIDS (2007)**	✓		

**Guidelines by the |Kenya National Council for Science & Technology 2004**	✓		

**Ethiopian National Health Research Ethics Guideline(2005)**	✓		

**Sudan**	✓		✓

**Guidelines for researchers and Ethics Review Committees in Zimbabwe by the Medical Research Council of Zimbabwe (2004)**	✓		✓

**Ugandan Guidelines by the National Council for Science & Technology 2006**	✓	✓	✓

**S. Africa MRC (2005)**	✓	✓	

**Nigeria National Code of Health Research(2006)**		✓	✓

**BRAZIL(1996)**		✓	✓

**India Council on Medical Research (ICMR) Code 2000**	✓	✓	

The provision of unavailable health care services and the support to local health care systems were not given as key benefits especially among the international guidelines such as DoH and CIOMS. Where these are recommended, provision of healthcare services encompasses gaining regular contact with health care workers and other measures that facilitate access to existing health care services. In addition, the benefits that are expected after research is complete mainly focus on the application of generalisable knowledge from proven interventions in their broadest sense. This can for instance be seen in the tone of Article 33 of the DoH (2008)^3^, Article 8 of CIOMS (2002) and UDBHR (2005) among others. It should however be noted that in most cases, the benefits in question are rather aspirational in the sense that they comprise the expected outcome of research, including generalisable knowledge.

### Who is the target beneficiary?

Three categories of beneficiaries are clearly evident from the guidelines. The first category comprises research participants; whether in the intervention or control group. Research participants are mainly expected to benefit from the intervention under investigation, and in some cases, they are assumed to be the bona fide beneficiaries of such interventions once they are proven to be effective as exemplified by the tone of some guidelines including the DoH, CIOMS, NBAC, NCOB and HUGO. The second category of beneficiaries is the community where research studies are undertaken or from which the participants are drawn. Special groups such as People Living with HIV and Aids (PLWHAs) or Men who have Sex with Men (MSM) are also included in this category. Guidelines that advocate for benefitting the host community and other special groups include; CIOMS (Epidemiology), HUGO, NBAC, and country specific guidelines such as those by India, Nigeria, Uganda and Brazil. Interestingly, guidelines by most developing countries focus on benefits to the community as opposed to the individuals, perhaps, pointing to the preferred category of beneficiaries in these settings. Such are likely to be in tension with most prominent international guidelines like the DoH, CIOMS, UNAIDS and to some extent the NCOB do not feature in this category.

The last group among beneficiaries is what might be referred to as the international community or society more generally. Often, this group is considered in relation to aspirational benefits that may accrue from accumulated generalisable knowledge. Such benefits include improved health and lifestyles resulting from a robust body of knowledge or availability of medicines to treat diseases afflicting the society. Table 3 shows the party (ies) expected to benefit by guideline.

### Obligation for providing benefits and mechanisms for making these decisions

There is little agreement amongst guidelines on how responsibility for providing benefits should be apportioned. Nonetheless, determination of responsibility appears to be based on how potential benefits are framed. For instance, when research benefits are seen as a compensation for risk assumed by participating in research, investigators and research sponsors are held responsible. This compensatory approach to delineation of responsibilities is mainly articulated by guidelines such as the CIOMS, NCOB, and the UNAIDS. Other guidelines that place responsibility on investigators and sponsors include the Declaration of Helsinki, the Indian Council for Medical Research, the Council of Europe (CoE) and the Ugandan national guidelines for medical research.

The scope of these obligations in terms of the beneficiaries and the period is however limited to those participating in research, and for the period before interventions are proven to be effective or introduced into the health care system. Some guidelines in this category especially those by Uganda and India, however, stretch this responsibility beyond benefits to individuals, to include provision of benefits to the host community. The Ugandan guideline for instance requires that investigators provide benefits while research is ongoing and that research should assist in securing access of proven interventions for the community. In both cases, the responsibilities of the sponsor are confined to financial support to aspects of the project and the provision of indemnity in the event of research related injuries.

On the other hand, when potential benefits are conceived from a social justice perspective including the duty not to exploit the vulnerable, responsibility for making them available is placed on all stakeholders involved in the research enterprise including communities, researchers, the host government and sponsors. Both the UNESCO declaration on human rights, the NCOB and the Nigerian Research Ethics Committee for instance place the responsibility to provide benefits on all stakeholders involved in the research enterprise, by emphasizing the importance of prior engagement among stakeholders to identify and apportion responsibility. Other guidelines that hold governments accountable for providing proven interventions into the healthcare system include the NCOB report, and the UNESCO declaration on bioethics and human rights.

Prior consultation among parties to be involved in research is the commonly recommended mechanism through which the extent and scope of researcher's responsibilities are to be determined. Most guidelines have specific clauses that point to the importance of prior engagement. Guideline 10 of the CIOMS 2002 for instance states that "before undertaking a study in a population or community with limited resources, the sponsor and the investigator must make every effort to ensure that first, the research is responsive to the health needs and priorities of the population or community in which it is to be carried out". Similarly, chapter 4.2 of the report by the NBAC 2001^4 ^recommend that "whenever possible, preceding the start of research, agreements should be negotiated by the relevant parties to make the effective interventions or other research benefits available to the host country after the study is completed". Other guidelines including the HUGO statement on benefit sharing, the NCOB report, and the DoH similarly recommend prior consultation among stakeholders to establish and agree on the benefits, who to benefit and the nature and extent of stakeholder responsibilities.

## Discussion

Ethical guidance relating to the norms that should govern global health research especially when undertaken in resource poor settings has come a long way. Their development has been characterised by a shift from a focus on protecting research participants from potential for exploitation, to that with a more holistic remit for securing community and host nation interests [2]. Most notable of the developments are those recommending community partnerships as avenues for delineating the social value of global health research in local settings. While these developments are welcome, danger signs are beginning to show and further progress in this area will need to engage with the root cause of the signs. For instance, while it is acknowledged that normative guidelines are meant to provide broad directives as opposed to prescription of specific actions, we have shown that there are disparities among guidelines regarding the responsibilities of researchers to participants and the community. With regard to the forms of benefits, some guidelines do not for instance consider access to research interventions as benefits, while in others these are clearly seen as such. Additionally, only a handful of guidelines recommend support to the health care system as a potential benefit while others clearly regard this as a key benefit. We are not suggesting that access to unproven research interventions or support to health care are necessary ingredients of a set of benefits from research but rather raising the issue that differing recommendations have obvious potential to result in differing practical interpretations of the ethical conduct of global health research.

Furthermore it is not clear how stakeholders can make decisions relating to the benefits, the party to benefit and the nature and extent of responsibilities for making these benefits available that would be ethically defensible after consulting all the research ethics guidelines identified. The tension arising from this lack of clarity is likely to be exacerbated by the fact that different stakeholders, if they have a choice, are likely to make decisions on their responsibilities on basis of the guidelines that most suit their preferences. This may be particularly likely given the potential financial implications for stakeholders funding research.

Interestingly, differences in emphasis regarding potential benefits and the beneficiaries are clearly evident between guidelines which perhaps represent the divide between "global opinion" and the views of particular countries within resource poor settings. Most international research ethics guidelines for instance recommend research participants as the key beneficiary, while those from resource poor settings demonstrate considerable interest in benefitting not only the participants, but also the trial community and even the host country (as shown in Table 3).

Lastly, there is some consensus among guidelines that the nature of the researcher's responsibilities be determined through prior engagement with local communities, the implication being that a practical mechanism exists to achieve this end. However, the likelihood of this process reaching consensus is questionable in the face of disagreement over benefits, the beneficiaries and by extension, the scope of obligations for providing these benefits. Indeed the appropriateness of prior engagement through such strategies as community consultation remain unclear judging from the debates relating to the difficulties of identifying the community to consult or even who the representatives are in cases where a 'community' is evident [25-28]. More practical challenges relate to the time appropriate for undertaking such consultations [25,27,29], and the feeling that community input to determine the nature of benefits is often not undertaken as consultation usually takes place only after the research protocol has been written and approved by the Institutional Review Boards (IRBs) [25,30]. The challenges faced by stakeholders who wish to undertake prior engagement should also be understood in the context of poorly functioning ethical governance systems often staffed with personnel ill-qualified to offer ethical guidance and contemplation [30-36]. Given these challenges and the different emphases in guidelines it therefore seems quite possible that different actors tackling the same research question but in different contexts might come up with very different conclusions with regard to benefits, beneficiaries, and obligations and thus very different perspectives on the social value of their research. Future developments within the ethics of global health research undertaken in resource poor settings will most likely benefit from further reflection on the broader ethical principles and from consideration of the practical realities of implementing these principles in real world context.

These findings should however be interpreted with some caution as documents provide only one window on the wider ethics discourse. It may not be possible to generalize to all low-income settings as most of the country specific guidelines reviewed were, by design, drawn from African countries with difficulties retrieving documents from a number of settings with the specific addition of the Indian and Brazilian guidelines. Were it to be possible, inclusion of more guidelines from the rest of the African settings would have provided a deeper context to our findings. In addition, we acknowledge that development of these guidelines was unlikely to be in isolation and there is potential for influence across countries and documents. No attempt was made to trace these interactions chronologically; something that may have helped put observed differences in context.

## Conclusion

Review of academic and policy literature in bioethics and the analysis of recommendations by research ethics guidelines point to a consensus that global health research undertaken in resource poor settings should be socially relevant and provide appropriate benefits if individual participants and populations are to be treated ethically. In theory consideration for benefit sharing is widely accepted as one of the means for promoting the social value of international collaborative health research although, as evidenced by intense debates within academic and policy literature in bioethics, there is not necessarily agreement on how benefit sharing should be approached. There is less consensus amongst major guidelines on the specific responsibilities of researchers over what is ethical in promoting the social value of research. Lack of consensus might have practical implications for efforts aimed at enhancing the social value of global health research undertaken in resource poor settings.

## Competing interests

The authors declare that they have no competing interests.

## Authors' contributions

All the authors participated in conceptualising the study. LGM undertook the review of guidelines and prepared the initial manuscript with substantial contribution from MP, RF and ME. All authors contributed to the development of the final manuscript and approved the final version.

## Appendix 1: Footnotes

^1 ^The National Institutes of Health adopts a three part definition of clinical research which includes; 1. Patient-oriented research. Research conducted with human subjects (or on material of human origin such as tissues, specimens, and cognitive phenomena) for which an investigator (or colleague) directly interacts with human subjects. Excluded from this definition are in vitro studies that utilize human tissues that cannot be linked to a living individual. Patient-oriented research includes: (a) mechanisms of human disease, (b) therapeutic interventions, (c) clinical trials, or (d) development of new technologies; 2. Epidemiologic and behavioral studies, 3. Outcomes research and health services research.

^2 ^Other sources including the compilation by the WHO were also visited including; http://www.who.int/ethics/research/en/index.html, databases on research ethics guidelines such as the one by Indiana University center for bioethics http://www.bioethics.iu.edu/body.cfm?id=87 and the global ethics observatory by UNESCO http://www.unesco.org/new/en/social-and-human-sciences/themes/global-ethics-observatory/access-geobs/

^3 ^As adopted during the 59th WMA General Assembly, Seoul, October 2008

^4 ^Available at http://bioethics.georgetown.edu/nbac/; Accessed 09.11.2011

## Pre-publication history

The pre-publication history for this paper can be accessed here:

http://www.biomedcentral.com/1472-6939/12/22/prepub

## Supplementary Material

Additional file 1 **Research ethics guidelines and policy documents reviewed**. Research ethics guidelines and policy documents on global health research. Brief summaries on the key research ethics guidelines and policy documents that have been developed to provide direction on matters relating to the protection of research participants and their communities when they participate in global health research. Click here for file
