# Supplementary material for: Ethics in practice: the state of the debate on promoting the social value of global health research in resource poor settings particularly Africa
Source: BMC Med Ethics. 2011 Nov 15;12:22. doi: 10.1186/1472-6939-12-22 (PMC3225323; doi:10.1186/1472-6939-12-22)
Supplement: Additional file 1 — Research ethics guidelines and policy documents reviewed. Research ethics guidelines and policy documents on global health research. Brief summaries on the key research ethics guidelines and policy documents that have been developed to provide direction on matters relating to the protection of research participants and their communities when they participate in global health research. [file 1472-6939-12-22-S1.PDF]

Additional File 1: Research ethics guidelines and policy documents reviewed

Guidelines	Contextual background
Declaration of Helsinki (2008)	The Declaration of Helsinki by the World Medical Association (WMA), a confederation of professional associations representing physicians which provides guidance to members through statements and declarations came into effect in 1964, to offer guidance to physicians on the ethical issues that arise while conducting biomedical research. The DoH was developed to address emergent problems that have arisen since the promulgation of the Nuremberg Code, and also to provide adequate guidance to help articulate already existing principles. Over time, the DoH has been taken as the fundamental document in research ethics, and has influenced the formulation of several other codes and guidelines especially those by developing countries.
CIOMS: International Guidelines for Biomedical Research Involving Human Subjects(2002)	The Council for International Organisations of Medical Sciences (CIOMS) is an international Non Governmental Organisation established jointly by WHO and UNESCO, which is meant to champion the interests of the biomedical and scientific community. The International guidelines for biomedical research involving human subjects by the CIOMS were developed to meet three goals; first, to offer guidance internationally when defining national policies on the ethics of biomedical research involving human subjects and secondly, to guide the domestication of ethical standards in local settings and thirdly to assist in improving the functioning of ethical review mechanisms.
The International Conference on Harmonization's Good Clinical Practice(1996)	The guidelines for good clinical practice (GCP) were released in 1996 as part of efforts by the International Conference on Harmonisation of Technical Requirements for Registration of Pharmaceuticals for Human Use (ICH). GCP guidelines reflect the "good" clinical practices of the European Union, Japan, the United States and those of Canada, the Nordic Countries and the World Health Organisation. The GCP require clinical trials to be conducted in accordance with ethical principles that have their origin in the DoH, and in conformity with applicable regulatory requirements. Essentially the ICH is aimed at providing technical standards for research sponsored by the pharmaceutical industry.
Report on the Ethical and Policy Issues in Research Involving Human Participants by *NBAC (2001)*	The National Bioethics Advisory Commission (NBAC) was established in 1995 through executive order by President Bill Clinton, with a threefold mandate; i) to provide advice and make recommendations to the National Science and Technology Council and other appropriate government bodies, on matters relating to programmes, policies, assignments guidelines, and regulations relating to bioethical issues arising from research on human biology and behaviour, ii) identify broad principles to govern the ethical conduct of research and iii) identify and provide advice on bioethical issues relevant to the National Science and Technology Council.

The Human Genome Organisation (HUGO) Statement on Benefit Sharing (2000)	The statement on benefit sharing was developed by the Human Genome Organisation's (HUGO) ethics committee, to address issues relating to whether and how benefits resulting from exploitation of human genetic research should be distributed. The statement outlines the underlying principles that needed to be taken into consideration while contemplating distribution of profits arising from commercial exploitation of genetic resources by governments, commercial enterprises or academic institutions.
The European Group on Ethics (EGE): Ethical Aspects of Clinical Research in Developing Countries (2003)	The European Group on Ethics (EGE) is an independent, pluralist and multidisciplinary body that provides opinions on ethical issues to the European Commission. The EGE advises the Commission on ethical questions relating to science and new technologies, either at the request of the Commission or on its own initiative. The Parliament and the Council may draw the Commission's attention to questions which they consider to be of major ethical importance. The EGE works closely with the Council of Europe (CoE), an intergovernmental organisation whose aims are to promote human rights protection and democracy in Europe. The activities of CoE cover health and education and develop conventions to offer guidance on its areas of influence. The Convention for the Protection of Human Rights and Dignity of Human Beings with regard to the application of Biology and Medicine (Convention on Human Rights and Biomedicine) contain relevant clauses on treatment of research participants. The guidelines on the 'Ethical Aspects of Clinical Research in Developing Countries' provides guidance when research is conducted in countries that are not party to the protocol.
Universal Declaration on Bioethics and Human Rights (2005)	The declaration by United Nations Educational, Scientific and Cultural Organisation (UNESCO) provides a universal framework of principles and procedures to guide states, individuals and other stakeholders in formulation of policies and other instruments and actions in the field of bioethics[1]. The declaration is meant to be domesticated into the relevant laws of UNESCO member countries and other international laws in conformity with human rights law.<br>The declaration was necessitated by the rise of transnational co operations in scientific activities and the need for universally shared values that represents this pluralism. The declaration uses the international human rights legislation as an essential framework and starting point for the development of bioethical principles such as human dignity, human rights and fundamental freedoms
Nuffield Council on Bioethics: Report on the ethics of research related to health care in developing	The Nuffield Council on Bioethics (NCOB) report addresses ethical issues that arise from externally funded health related research undertaken in resource poor settings. The report was developed to offer a unified ethical framework that unifies the divergent and conflicting guidance faced by those involved in externally sponsored research in resource poor settings, and assist those involved in developing national guidance for ethical review of research. The NCOB report

[1] Bioethics is defined within the UNESCO explanatory notes on the elaboration of the preliminary draft declaration on universal norms as "a systematic, pluralistic and interdisciplinary field of study involving the theoretical and practical moral issues raised by medicine and life sciences as applied to human beings and humanity's relationship with the biosphere".

countries (2005)	does not set out guidelines for the conduct of research, but the ethical framework presented is expected to be relevant to a wide range of organisations, agencies, national governments and people participating in research.
UNAIDS/WHO guidance document on Ethical Considerations in biomedical HIV prevention trials (2010)	The current guidance is a revised version of the guidelines issued in 2000. The revised version was meant to address the new development and changes in the development of HIV prevention and vaccine trials, including the need for creating effective partnerships, collaboration and community participation, inclusion of adolescents in research, gender considerations, provision of support, care and treatment to participants and communities and post trial responsibilities of actors involved in research. The guideline suggests standards and processes for arriving at standards to inform discussion at local and international level for the conduct of biomedical HIV prevention trials. These standards and processes are articulated through nineteen (19) guidance points. The revised guideline is further informed by other international codes including the Nuremberg Code, DoH, CIOMS, the ICH, GCP and the UNAIDS Interim Guidelines on Protecting the Confidentiality and Security of HIV Information (2007)
National Council for Science and Technology: Guidelines for Ethical Conduct of Biomedical Research in Kenya (2004)	The Kenyan guidelines were specifically formulated to offer direction regarding the conduct of health related research. Although it is noted in the preamble that the guidelines draw from international guidelines such as the DoH and the CIOMS, are mainly formulated alongside the unified framework for determining whether research is ethical proposed by Emanuel and colleagues. Interestingly however, the guidelines contain separate ethical obligations for externally sponsored research. These obligations mainly address issues relating to requirements for ethical approval, responsiveness to local health needs, reasonable availability and benefits of such collaboration.
Uganda National Council for Science and Technology: National Guidelines for Research Involving Humans as Research Participants (2006)	The current guidelines were revised from the 1997 version. The revisions were undertaken to reflect the current norms of scientific research practice and ethical conduct. In addition, the scope of the guidelines was broadened to include all research involving humans[2] as subjects including research in social sciences and humanities, alternative medicines and those involving use of stored human biological materials. These guidelines specifically addresses the ethical issues arising from increased international collaborative research in resource poor settings, aiming to present a national framework for harnessing the benefits associated with increased research while protecting the rights, interests, values and welfare of the research participants and communities.
South African Medical Research Council: Guidelines on Ethics for Medical Research	The MRC guidelines on medical research comprise a series of five books. Book one, contains general principles for medical research while book two and three presents guidelines on reproductive biology & genetic research, and use of animals respectively. Book four covers guidelines on the use of Biohazards and Radiation, while the last book presents ethical guidelines

[2] According to the Ugandan guidelines, research involving human subjects includes; i) clinical investigations, that is, any experiments or study on one or more persons which involves a test product/article, whether a drug, treatment, procedure, or device; ii) Social-behavioral studies which involve interaction with or observation of people; and iii) basic scientific research to study the biology or persons or organs and specimens thereof; iv) Systematic collection, storage and analysis of data on humans.

(2005)	for research on HIV Vaccine trials. The guidelines are further informed by the international debates relating to the general principles that govern human subjects' research and those considerations that are particular to resource poor settings. The MRC guidance is firmly built into the South African constitution, which gives them legislative powers.
National Code of Health Research Ethics: National Health Research Ethics Committee of Nigeria (2006	The Nigerian code of ethics is established through the National Health Bill (2004) to offer guidance on the conduct of health related research as well as the establishment and mandate of research ethics committees. The code applies to all health research involving human participants, conducted, supported or otherwise subject to regulation by any institution in Nigeria, except; i) research on regular and special educational strategies or that which is aimed at establishing the effectiveness of instructional techniques, curricula or classroom management methods, provided this is undertaken within the established educational settings; ii) research involving use of educational tests (cognitive, diagnostic, aptitude, achievement) survey procedures, interviews or observation of public behaviour.[3] Other exceptions include; iii) research involving collection of existing data, documents, records or pathological specimen; iv) studies that are meant to evaluate the outcome of procedures, programmes and services (quality improvement), since these already evaluate procedures that are deemed to be standard practice; v) innovative or non validated medical treatment, that is solely designed for the benefit of the patient. Such exceptions are however determined by the Health Research Ethics Committee (HREC).
Sudan (2008)	The National Guidelines for Ethical Conduct of Research Involving Human Subjects was issued in 2008 by the Directorate General of Health Planning and Research, of the National Ministry of Health. The guideline covers both medical and behavioural studies pertaining to human health as well as other forms of research involving human participants including social sciences, behavioural and epidemiological research.<br>The guideline is based on international research ethics guidelines including those by CIOMS, and DoH by WMA
Guidelines for researchers and Ethics Review Committees in Zimbabwe by the Medical Research Council of Zimbabwe (2004)	The Medical Research Council of Zimbabwe also doubles up as the National Ethics Committee (NEC), and part of its specific tasks is to provide ethical guidance on all types of research undertaken in Zimbabwe. The MRCZ is guided by the ethical principles espoused i the Declaration of Helsinki and the CIOMS and other applicable international and national research guidance documents.
Ethiopian National	The Ethiopian Science and Technology Commission is a federal body tasked with guiding,

[3] Provided the information is recorded in non identifiable manner and that any disclosure of the participants responses cannot place the participant at risk of criminal or civil liability, or harmful to a person's financial standing, employability or reputation

Health Research Ethics Review Guideline: National Health Science & Technology Council (2005)	coordinating and facilitating all science and technology activities including health. The current National Health Research Ethics Review Guideline was issued in 1995, and revised in 1997 and 2004, to reflect the new developments in health research and attendant ethical issues arising from biomedical, clinical and epidemiological studies.<br>The guideline draws from international codes including the Nuremberg Code, Belmont Report, the ICH, Declaration of Helsinki and the CIOMS.
Indian Council of Medical Research (2006)	The ethical guidelines for biomedical research on Human Subjects by the Indian Council of Medical Research (New Delhi) were for instance promulgated in response to biotechnology research ushered in by advances in science and technology. The ICMR broadly draws from international codes developed by organisations such as the WMA, CIOMS and the WHO.<br>The ICMR resolution aims to provide guidance to all types of research and therefore contains a set of specific principles to be applied within clinical trials of drugs or vaccines, epidemiological studies, human genetic research, research into transplantation and assisted reproductive technologies.
Brazilian Resolution of Medical Research (304/2000)	The Brazilian Code/Resolution of medical research is largely based on the main international documents such as the Nuremberg code, the Declaration of Helsinki and the guidelines by the CIOMS and related Brazilian legislation. In addition, the resolution is based on the principles of autonomy, non maleficence, beneficence and justice, and is aimed at promoting the rights and duties of the scientific community, the research participants and the state.
